# Influence of the Maillard Reaction on Properties of Air-Assisted Electrospun Gelatin/Zein/Glucose Nanofibers

**DOI:** 10.3390/foods12030451

**Published:** 2023-01-18

**Authors:** Songqi Liu, Shiyuan Luo, Yuanli Li, Huange Zhang, Zhihe Yuan, Longchen Shang, Lingli Deng

**Affiliations:** College of Biological and Food Engineering, Hubei Minzu University, Enshi 445000, China

**Keywords:** air-assisted electrospinning, gelatin, zein, Maillard reaction, glucose

## Abstract

To develop biodegradable, sustainable, and environment-friendly functional food-packaging materials, gelatin/zein/glucose nanofibers were fabricated through air-assisted electrospinning and then crosslinked by the Maillard reaction under mild conditions (60 °C and 50% relative humidity) in this study. Compared to traditional electrospinning, air-assisted electrospinning increased the yield of nanofibers by 10 times, and the average diameter from 263 nm to 664 nm, while the airflow facilitated uniform and smooth nanofiber formation. During the Maillard reaction in 0–5 days, the gelatin/zein/glucose showed no morphology change. Fourier transform infrared spectra analysis indicated that gelatin interacted with zein through hydrogen bonding and the occurrence of the Maillard reaction among the protein and glucose molecules. After four days of Maillard reaction, the nanofibers presented higher thermal stability, the most hydrophobic surface (water contact angle: 133.6°), and stiffer network structure (elastic modulus of 38.63 MPa, tensile strength of 0.85 MPa). Overall, Maillard-reaction-crosslinked gelatin/zein/glucose nanofibers showed favorable physical properties, which suggests their potential for application in food-active packaging.

## 1. Introduction

Functionalized electrospun nanofibers possess multiple attributes, e.g., moderate preparation conditions, high porosities, high surface/volume ratio, outstanding mechanical properties and acceptable morphologies, and so on, which provide excellent prospects for application in the field of food. Currently, many studies mainly focus on antioxidant packages [1], antimicrobial packages [2], embedding release [3], enzyme immobilization [4], rapid testing [5], etc. The formation of electrospun nanofibers is closely related to the polymer matrix (such as optimum conductivity, viscosity, and surface tension) [6]. In recent years, food hydrocolloids such as polysaccharides and proteins have received more and more attention than synthetic polymers due to biodegradability, sustainability, and environmental-friendliness considerations.

Gelatin is a water-soluble protein derived from animal tissue, while zein is a hydrophobic storage protein from corn that is highly biocompatible and exhibits film-forming properties [7,8]. Thus far, a few studies have demonstrated that gelatin and zein can be electrospun. As an example, Ansarifar and Moradinezhad [9] prepared zein nanofibers loaded with thyme essential oil to preserve strawberries; these nanofibers were superbly linear, exhibited smooth surfaces, and were devoid of beads. Uniform and smooth gelatin nanofibers containing eugenol were fabricated by Li et al. [10]. However, mechanical strength and long-term solvent resistance remain to be improved to mimic food package criteria in practical applications. Crosslinking is considered a good technique for modifying materials, and chemical crosslinking with glutaraldehyde and genipin has been well-studied thus far [11,12], but problems associated with toxic residues limit their applicability in the food industry.

The Maillard reaction, which involves grafting the amino groups of a protein with the carbonyl moieties of a reducing sugar, has been reported to offer a green chemical crosslinking strategy [13]. Kchaou et al. [14] prepared fish gelatin films crosslinked with glucose by the Maillard reaction at 90–130 °C and found that water solubility, wettability, and antioxidant activity were improved remarkably. Moreover, there were significant differences in nanofiber properties between different treatment temperatures, with the best performance in all aspects at 90 °C. Similarly, Kwak et al. [15] claimed mechanical and antioxidant properties of glucose- or fructose-crosslinked gelatin nanofibers were improved. Previous research revealed that electrospun gelatin/zein nanofibers crosslinked by glucose (3 h at 140 °C) were highly wettable, biocompatible, and exhibited good mechanical properties [16]. However, high temperatures afford conjugates that exhibit irreversible loss of functionality, as well as a lack of control over unwanted Maillard reaction products [17]. High temperature may also damage the encapsulated bioactive components in nanofibers, which has limited the application of high-temperature-glycated nanofibers on active packaging. Consequently, a mild Maillard reaction condition was considered in this study.

In addition, the low yield afforded by traditional electrospinning is another obstacle to the use of nanofibers in the food industry. Air-assisted electrospinning technology is considered to have excellent application prospects, and adding airflow forces based on traditional electrospinning technology has been shown to improve performance and significantly increase yield [18,19]. Aminyan and Bazgir [18] and Li et al. [20] prepared, respectively, nanofibrous superabsorbent and polytetrafluoroethylene ultrafine fibrous porous membranes. Compared to traditional electrospinning, nanofibers exhibited excellent flexibility, uniformity, continuity, and flawlessness. Duan and Greiner [19] optimized flow rate and air-blowing rate during electrospinning, which increased productivity to the range of 1.16–3.60 g/h compared to that associated with traditional electrospinning (<1 g/h).

This study aimed to fabricate gelatin/zein/glucose nanofibers through air-assisted electrospinning, with subsequent crosslinking by the Maillard reaction under mild conditions. We hypothesized that gelatin/zein nanofiber films with physical properties suitable for food applications will be produced by the Maillard reaction under mild conditions. Scanning electron microscopy (SEM) was used to evaluate the microscopic morphology and diameter distribution of nanofibers. To interpret changes in macroscopic performance such as the water contact angle (WCA), mechanical property, water vapor permeability (WVP), and water stability, the interaction between protein and glucose was studied using Fourier transform infrared (FTIR), X-ray photoelectron spectroscopy (XPS), and thermal analysis techniques.

## 2. Materials and Methods

### 2.1. Chemicals

Gelatin (Type B, Bloom 250, MW∼100 kDa), glucose, and acetic acid were purchased from Aladdin Reagent Database Inc (Shanghai, China). Zein (grade Z3625, 22–24 kDa) was purchased from Sigma Aldrich (St. Louis, MO, USA).

### 2.2. Solution Preparation

Gelatin (15% *w/v*) and zein (15% *w/v*) were dissolved in 80% aqueous acetic acid. Glucose (5% *w/v*) was then added to the solution in accordance with our previous report [16]. To guarantee full dissolution, all samples were agitated throughout the night.

### 2.3. Air-Assisted Electrospinning

**Figure 1:**
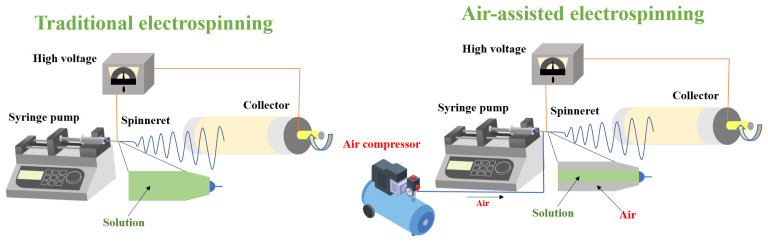
Schematic showing the traditional and air-assisted electrospinning setups.

A schematic illustration of traditional electrospinning and air-assisted electrospinning is shown in Figure 1. To transfer each solution to the needle tip (with an inner diameter of 0.5 mm), a 10 mL syringe was used and pushed through the pressure pump at a rate of 10.0 mL/h for air-assisted electrospinning and a rate of 1.0 mL/h for traditional electrospinning. To create a high-voltage direct-current (DC) electric field, a high-voltage power generator was employed. Air pumps were then used to maintain a 400 L/h airflow rate. With a tip-to-collector distance of 15 cm, the electrospinning voltage was adjusted to 18 kV.

### 2.4. Maillard Reaction

The Maillard reaction was conducted in a heat- and relative humidity-controlled chamber (DHTHM-16-0-P-SD, Doaho Test Co., Ltd., Shanghai, China) at 60 °C and 50% relative humidity (RH) for 0, 1, 2, 3, 4, and 5 days.

### 2.5. Fiber Morphologies

Vacuum gold spraying was applied to treat the fibers, and SEM was utilized to evaluate their microscopic morphology (SU8010, Hitachi, Tokyo, Japan). Using Nano Measurer 1.2, the diameters of forty randomly selected fibers were determined from the SEM images, and the related diameter distribution was estimated and fitted.

### 2.6. Color Analysis

The colorimetric a* (redness) and b* (yellowness) values of the fiber films were measured using a chroma meter (CS-820N, Hangzhou CHNSpec Technology Co., Ltd., Hangzhou, China).

### 2.7. Infrared Spectroscopy

Using a Fourier transform infrared (FTIR) spectrometer, infrared spectra of a tiny sample taken from the nanofibers were captured (scanning range: 4000–400 cm^−1^; resolution: 2 cm^−1^; number of scans: 32). Prior to each scan, air was used as the background and subtracted. The infrared spectra of nanofibers crosslinked for 0–5 days were compared quantitatively by calculating the relative absorbance changes (∆RA). The absorbance of the peak was used divided by the sum of the absorbance of all the peaks detected in the spectrum [21].

### 2.8. Thermal Properties Analysis

With differential scanning calorimetry (DSC) and thermogravimetric analysis (TGA) (NETZSCH-Gerätebau GmbH, Selb, Germany), the thermal characteristics of the nanofibers were identified. Each sample was precisely weighed at 6–10 mg and sealed in an aluminum crucible. The sample was then heated under dry N_2_ from 30 to 600 °C at 10 °C/min. The same conditions were used while using empty crucibles as references.

### 2.9. Surface Elemental Analysis

XPS (AXIS SUPRA, Kratos Analytical Inc., Manchester, UK) was used to determine the chemical state and composition of the nanofiber surface. The spectrum survey scans were obtained over the 0–1350 eV binding energy range at a detector pass energy of 100 eV and high-resolution spectra were recorded for the C1s region at pass energy of 50 eV. The spectra were processed using Advantage software (Thermo Scientific, East Grinstead, UK) and OriginPro 2022b (OriginLab Corporation, Northampton, MA, USA) [22].

### 2.10. Water Contact Angle Tests

The WCA of the nanofibers was carried out using a tensiometer (OCA 20, Dataphysics Instruments, Filderstadt, Germany). A droplet of liquid (3 µL) was deposited on the film surface (20 × 40 × 0.2 mm) with a precision syringe, and the contact angles were measured at 5 s after the drop contacted the nanofibers.

### 2.11. Mechanical Properties

Using a DR-508A (Dongri Instrument Ltd., Dongguan, China) computer tensile testing machine, the tensile strength (TS), elastic modulus (EM), and elongation at break (EB) were calculated. The nanofibers, which were around 0.1 mm thick, were sliced into 5 × 1.5 cm pieces and put in a fixture, after which the thickness, width, and intercept of the samples were precisely measured, entered into the software, and then subjected to a tensile load of 5 N and a tensile rate of 5 mm/min. Each sample was measured five times. Following are the calculations for the TS, EM, and EB [23]:

(1) $$\mathrm{Tensile}\;\mathrm{strength}\;\left(\mathrm{MPa}\right)=\frac{\mathrm{Load}\;\mathrm{at}\;\mathrm{Break}}{\mathrm{Original}\;\mathrm{width}\;\times \;\mathrm{Original}\;\mathrm{thickness}}$$

(2) $$\mathrm{Elongation}\;\mathrm{at}\;\mathrm{break}\;\left(\%\right)=\frac{\mathrm{Elongation}\;\mathrm{at}\;\mathrm{rupture}}{\mathrm{Original}\;\mathrm{test}\;\mathrm{length}}\;\times 100$$

(3) $$\mathrm{Elastic}\;\mathrm{modulus}\;\left(\mathrm{MPa}\right)=\frac{\mathrm{Stress}}{\mathrm{Strain}}$$

### 2.12. Water Vapor Permeability

The lip of a 10 mL water-permeable cup was sealed with a sheet of uniformly thick, 6 cm diameter nanofiber film, and the cup was then placed in a desiccator. Hourly weights and records were kept for all samples. This process was repeated three times over 6 h. WVP was calculated as [24]:

(4) $$\mathrm{WVP}(g/m\cdot s\cdot \mathrm{pa})=\frac{W_{s}}{\mathrm{At}\;}\times \frac{L}{\Delta P}$$

where A is the contact area between the nanofibers and water vapor (cm^2^); L is the thickness (cm); ∆P is the rated vapor pressure differential (Pa) (2237.8 Pa, 28 °C); W_s_/t is the linear regression of weight over time (g/s).

### 2.13. Stability to Water

Nanofibers (5 mg) were soaked in water for two months and then vacuum freeze-dried (LGJ-10 Beijing Songyuan Huaxing Technology Development Co., Ltd., Beijing, China). The microscopic morphologies of the fibers were observed by SEM.

### 2.14. Statistical Analysis

The quantitative data were statistically analyzed and expressed as means ± standard deviations. The statistical significance of mean values between multiple treatment groups was accessed by one-way analysis of variance (ANOVA) with Tukey’s tests using the OriginPro 2022b (OriginLab Corporation, Northampton, MA, USA). A *p*-value < 0.05 was considered statistically significant.

## 3. Results and Discussion

### 3.1. Fiber Morphologies

**Figure 2:**
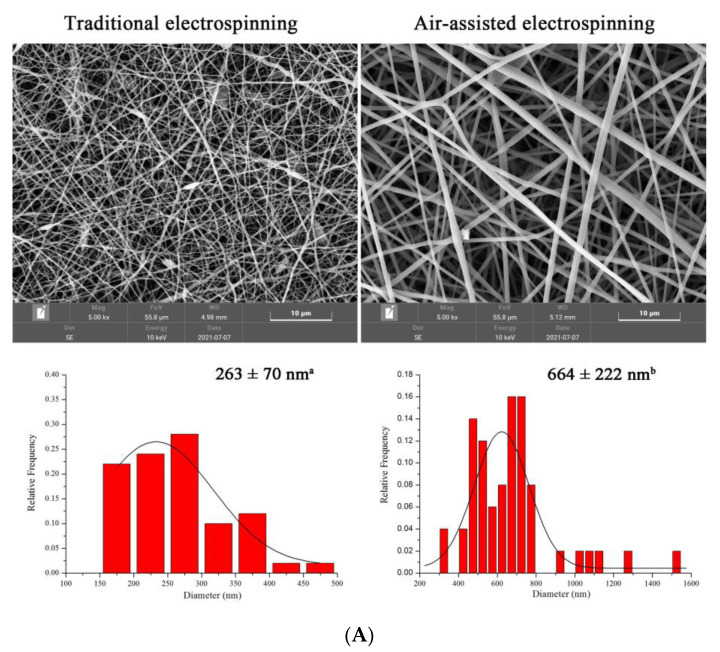

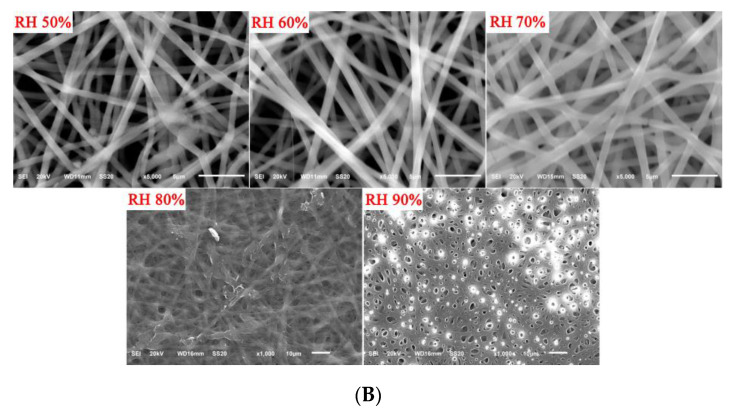
(**A**) SEM images and fiber diameter distributions of the gelatin/zein/glucose nanofibers fabricated by traditional electrospinning and air-assisted electrospinning (values with different superscript letters are significantly different, *p* < 0.05). (**B**) SEM images of gelatin/zein/glucose nanofibers prepared at 60 °C and 50–90% RH for 24 h.

SEM images of gelatin/zein/glucose nanofibers fabricated by traditional and air-assisted electrospinning are presented in Figure 2A. Compared with the traditional electrospinning, air-assisted electrospinning facilitated the formation of smooth and uniform nanofiber devoid of beads, with nanofiber diameter observed to increase from 263 ± 70 to 664 ± 222 nm when air-assisted. Even though some study reported lower mean diameter of nanofiber fabricated by air-assisted electrospinning compared to that of traditional electrospinning, the variance in the electrospinning parameters should not be neglected. Shi et al. [25] prepared polytetrafluoroethylene (PTFE) ultrafine fibrous porous membranes via electrospinning, solution blow spinning, and air-assisted electrospinning with consistent flow rate. The PTFE nanofiber membranes generated through air-assisted electrospinning had lower mean diameter and pore size than those prepared through traditional electrospinning, while the collector distance was much higher for air-assisted electrospinning (800 mm) compared to that of traditional electrospinning (200 mm). It is well-known that the flow rate has a positive linear relationship with fiber diameter [26], and an increase in diameter is inevitable as the flow rate increases from 1 to 10 mL/h when other electrospinning parameters remained the same. The observed differences in fiber morphologies and diameter are related to the airflow; airflow has a stable stretching effect on the polymer solution that increases the solution flow rate [19]. The high airflow velocity accelerates fiber deflection toward the deposition receiver and solvent evaporation, with a larger nanofiber diameter and an improved morphological structure obtained as a result [27]. In addition, electrospinning efficiency is closely related to the flow rate, which was observed to increase from 1 to 10 mL/h in this study, with a concomitant 0.3 to 3 g/h increase in yield [19], indicating that air-assisted electrospinning is a potential technology for the large-scale preparation of nanofibers.

SEM images of electrospun gelatin/zein/glucose films obtained after the nanofibers had been subjected to the Maillard reaction at 60 °C for 24 h at various RH are presented in Figure 2B. With an increase in relative humidity from 50% to 90%, the nanofibers gradually appeared to dissolve and swell, and started to lose fiber structure after the relative humidity reached 80%. The practical application value was low, so 50% was selected for the follow-up study.

**Figure 3:**
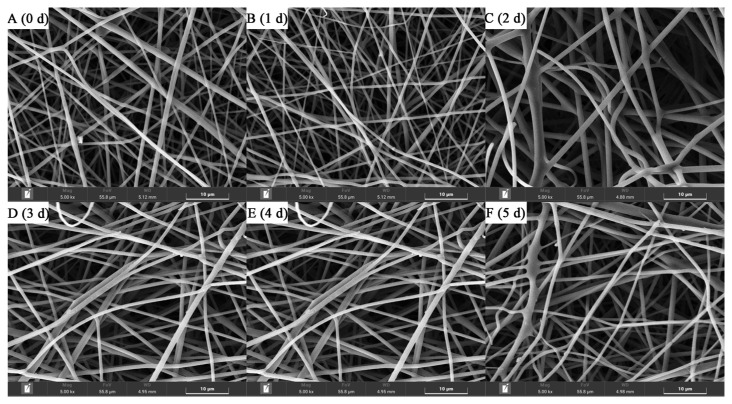
Morphologies of gelatin/zein/glucose nanofiber films after Maillard reactions conducted at 60 °C and 50% RH for 0 (**A**), 1 (**B**), 2 (**C**), 3 (**D**), 4 (**E**), and 5 days (**F**).

**Figure 4:**
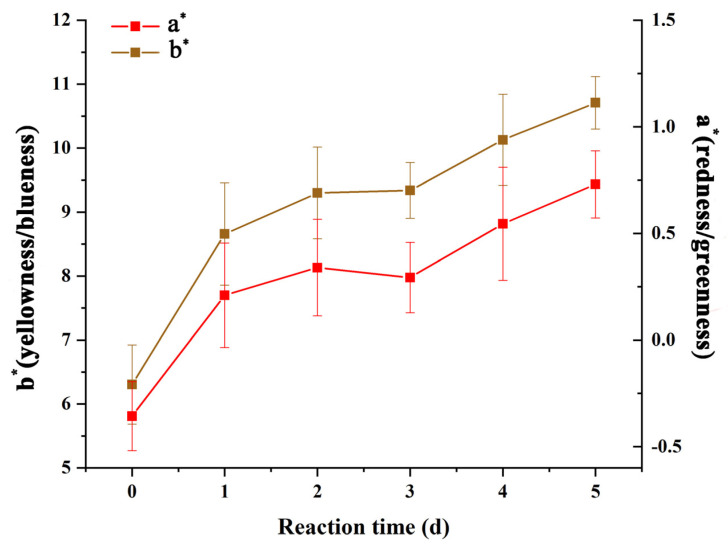
Chromaticity a* and b* values of gelatin/zein/glucose nanofiber films after Maillard reactions conducted at 60 °C and 50% RH for 0~5 days.

Figure 3 shows the morphology of gelatin/zein/glucose nanofibers after Maillard reaction at 60 °C and 50% RH for 0~5 days. While the nanofiber diameters were not significantly affected by the reaction time, small sections of various nanofibers appeared to dissolve and swell through water absorption with increasing reaction time. Figure 4 shows the chromaticity a* (red–green) and b* (yellow–blue) values of the nanofibers after Maillard reaction, which appeared to increase remarkably on days 1–3 compared to day 0 owing to Schiff-base formation [28]. Then, a* and b* values increased continuously on days 4–5 due to the formation of yellowish intermediates in the intermediate stage of the Maillard reaction.

### 3.2. Infrared Spectroscopy

**Figure 5:**
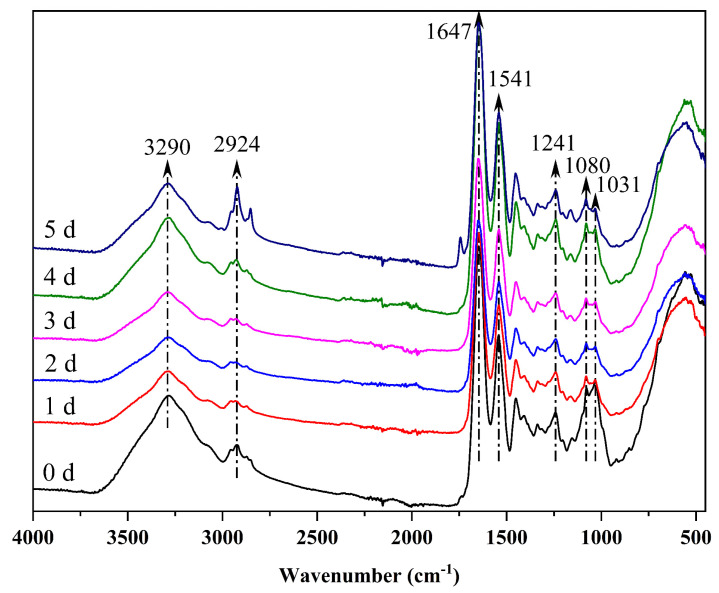
FTIR spectra of gelatin/zein/glucose nanofibers after Maillard reactions conducted at 60 °C and 50% RH for 0~5 days.

Figure 5 presents the FTIR spectra of gelatin/zein/glucose films after the nanofiber films had been subjected to Maillard reactions for 0~5 days. The band centered at 3290 cm^−1^ was assigned to the O–H and N–H stretching vibrations that may be associated with hydrogen bonding interactions between gelatin and zein [29]. The band at approximately 2924 cm^−1^ was attributed to the –CH_2_ stretching vibration of aliphatic groups [30]. The absorption peaks near 1647 cm^−1^, 1541 cm^−1^, and 1241 cm^−1^ were ascribed to the C=O stretching vibrations of the amide I, the C–N stretching vibration of amide II, and C–N stretching and N–H bending combination of amide III, respectively [16]. The peaks at 1080 cm^−1^ and 1031 cm^−1^ resulted from the C–O stretching of glucose [31]. The protein is uniformly dispersed in the nanofibers due to the absence of peak split in the spectra [32].

Relative absorbance of the peaks after Maillard reaction for 0~5 days is listed in Table 1. The initial stage of the Maillard reaction involves glycation. The condensation reaction between the carbonyl group of glucose and the free amino group (mainly the ε-amino groups of lysine) of the protein dehydrated to form a Schiff base, which formed an Amadori product after automatic rearrangement [33]. After crosslinking, the relative absorbance of amide I (1647 cm^−1^) and amide II (1541 cm^−1^) were significantly enhanced, and the peaks (1080 cm^−1^ and 1031 cm^−1^) representing the C-O stretching of glucose were reduced considerably, to prove the occurrence of glycation.

### 3.3. Thermal Analysis

**Figure 6:**
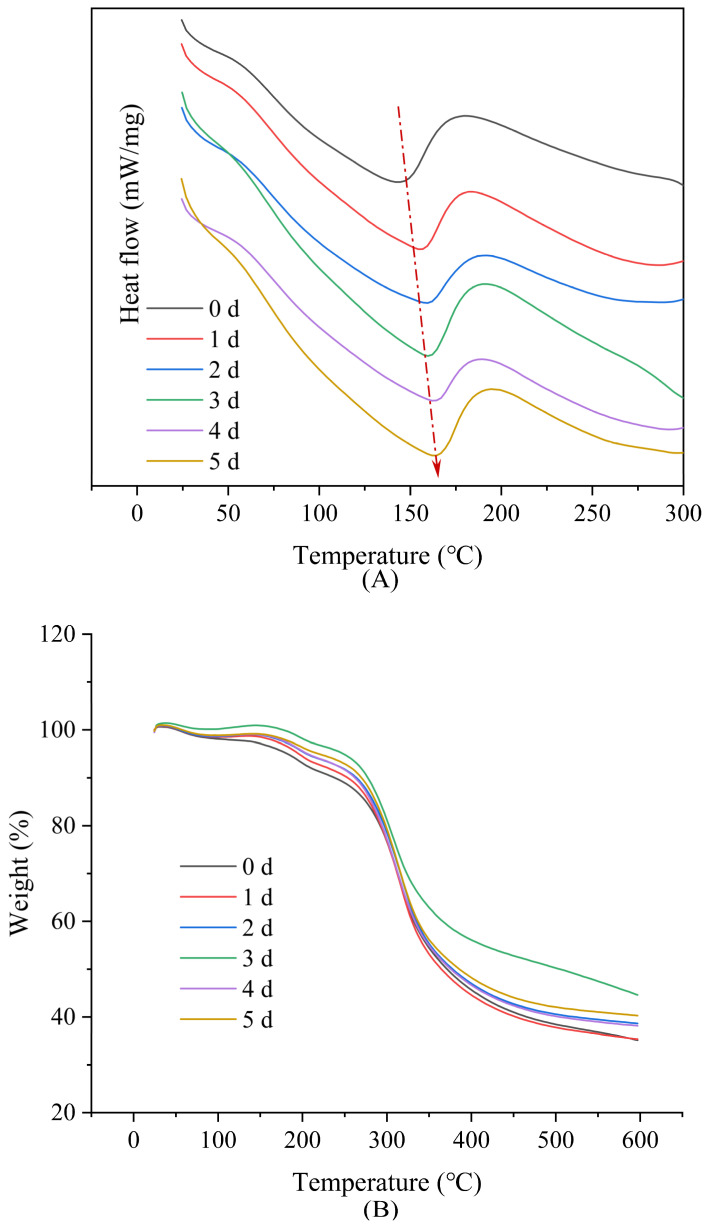

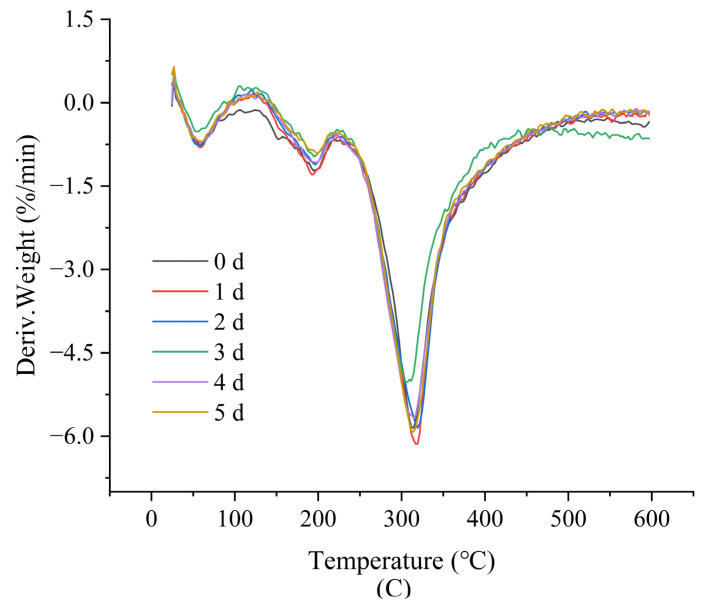
The DSC curves (**A**), TGA weight loss curves (**B**), and derivative weigh loss curves (**C**) of gelatin/zein/glucose nanofibers after Maillard reactions at 60 °C and relative humidity of 50% for 1~5 days.

The DSC and TGA curves of the films after Maillard reaction for 0~5 days are presented in Figure 6, with results summarized in Table 2. The characteristic heat absorption peaks and the corresponding enthalpy of the DSC curves are referred to as the denaturation temperature (T) and denaturation enthalpy (ΔH), respectively. The denaturation temperature rapidly increased from 143.5 °C to 155.4 °C during 1 day of the Maillard reaction, with a value of 163.9 °C observed by day 5. The increase in denaturation temperature implied an increase in the thermal denaturation stability of the films, suggesting the formation of a more stable conjugate compound between the protein and glucose. Kchaou et al. [34] likewise confirmed that crosslinking between fish gelatin and protein led to a more thermally stable matrix.

As shown in Figure 6B,C and Table 2, the degradation of nanofibers was mainly divided into two steps. The initial step (3–10% weight loss) was mainly attributable to polymer-network degradation (~200 °C), while most of the nanofiber weight was lost in the second step, with residue (>600 °C) attributable to inorganic compounds derived from the thermal degradation of the raw materials [35,36]. Crosslinked nanofibers showed higher onset decomposition temperature (peak 1) and lower weight loss, indicating higher thermal stability.

### 3.4. Water Contact Angle Analysis

**Figure 7:**
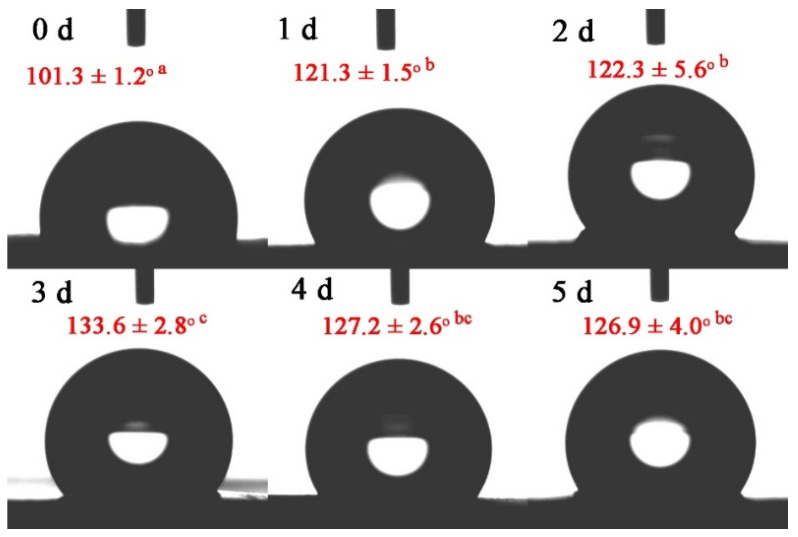
The water contact angles of gelatin/zein/glucose nanofibers after Maillard reactions conducted at 60 °C and 50% RH for 0~5 days. Values with different superscript letters are significantly different (*p* < 0.05).

The WCA of the nanofibers after Maillard reaction for 0~5 days is presented in Figure 7. The WCA changed from 101.3 ± 1.2° to 133.6 ± 2.8° at 0–3 days. Zein is highly hydrophobic, resulting from the presence of non-polar amino acids (proline and glutamine) [37]. When gelatin and zein were co-spun in equal proportions, the intermolecular hydrogen bonding interactions reached their maximum, resulting in more non-polar groups facing outward and exhibiting hydrophobicity, and then after glycation, the outward-facing polar groups continued to decrease. Therefore, hydrophobicity increased. The WCA decreased to 126.9 ± 4.0° on the fifth day, which was derived from degradation of the Amadori/Heyns product in the intermediate step of the Maillard reaction, where free amino groups are produced. Therefore, hydrophobicity tended to decrease.

### 3.5. Surface Elemental Analysis

**Figure 8:**
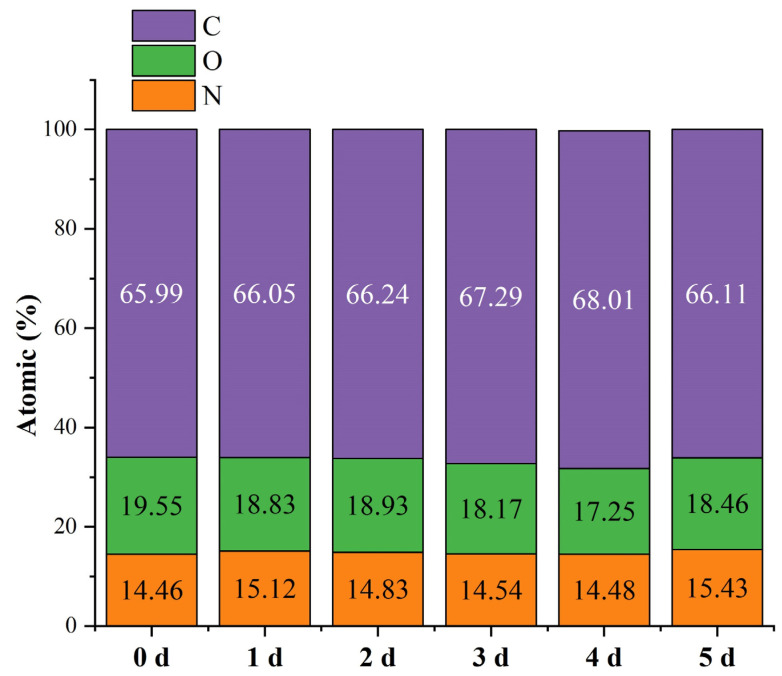
Elemental compositions (atomic%) of the gelatin/zein/glucose nanofiber film surfaces after Maillard reactions conducted at 60 °C and 50% RH for 0~5 days.

The elemental compositions of the nanofiber surfaces were determined by XPS, the results of which are shown in Figure 8. The C, O, and N atomic contents were significantly different after the Maillard reaction; the proportion of C increased from 65.99% to 68.01% during the first four days of the reaction, while the proportion of O decreased from 19.55% to 17.25%, with no significant change in the N proportion observed. Such changes in atomic content were considered to signal the onset of the Maillard reaction [38]. The decrease in O content might be related to the water loss during crosslinking. However, C and O contents of 66.11% and 18.46% were observed by the fifth day, with the N content increasing from 14.48% to 15.43% over this period. The elevated N content was associated with the degradation of the Amadori/Heyns products in the intermediate step of the Maillard reaction, which produced free amino acids, whose hydrophilicity might change O. This conclusion was consistent with the WCA results.

**Figure 9:**
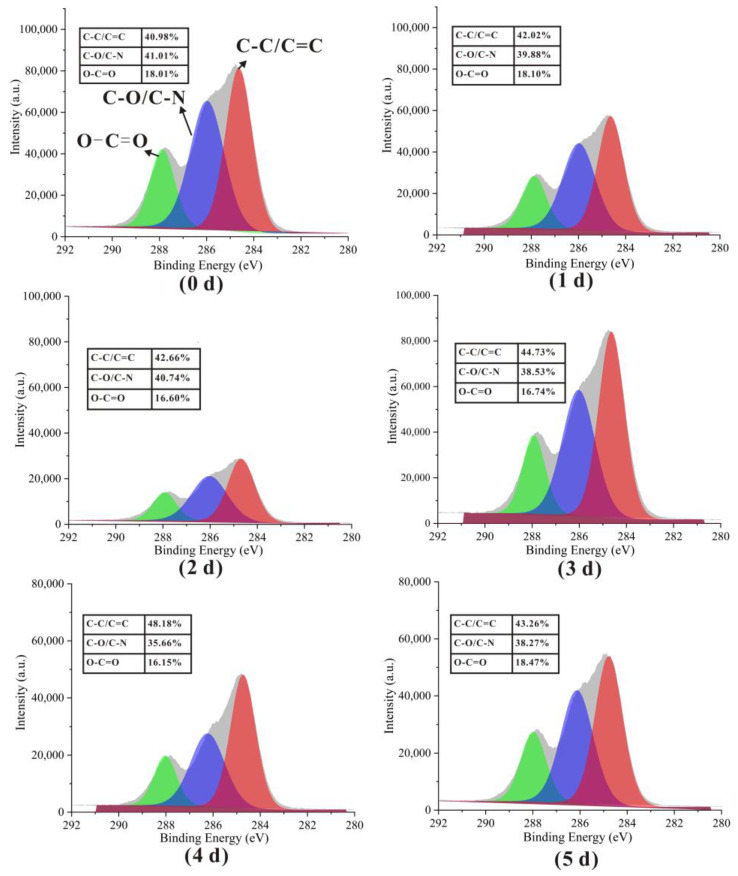
High-resolution C1s XPS spectra of gelatin/zein/glucose nanofibers after Maillard reactions conducted at 60 °C and 50% RH for 0~5 days.

The three characteristic peaks observed at 284.7, 286.2, and 288.0 eV in the high-resolution C1s spectra in Figure 9 correspond to C–C/C=C, C–O/C–N, and O–C=O, respectively [39]. The C–C/C=C proportion was observed to increase from 40.98% to 43.26%, while C–O/C–N and O–C=O decreased from 41.01% to 35.66% and 18.01% decreased to 16.15%, respectively, over the first four days; these trends appeared to pick up by the fifth day, consistent with the occurrence of Maillard reactions.

### 3.6. Mechanical Property Analysis

**Figure 10:**
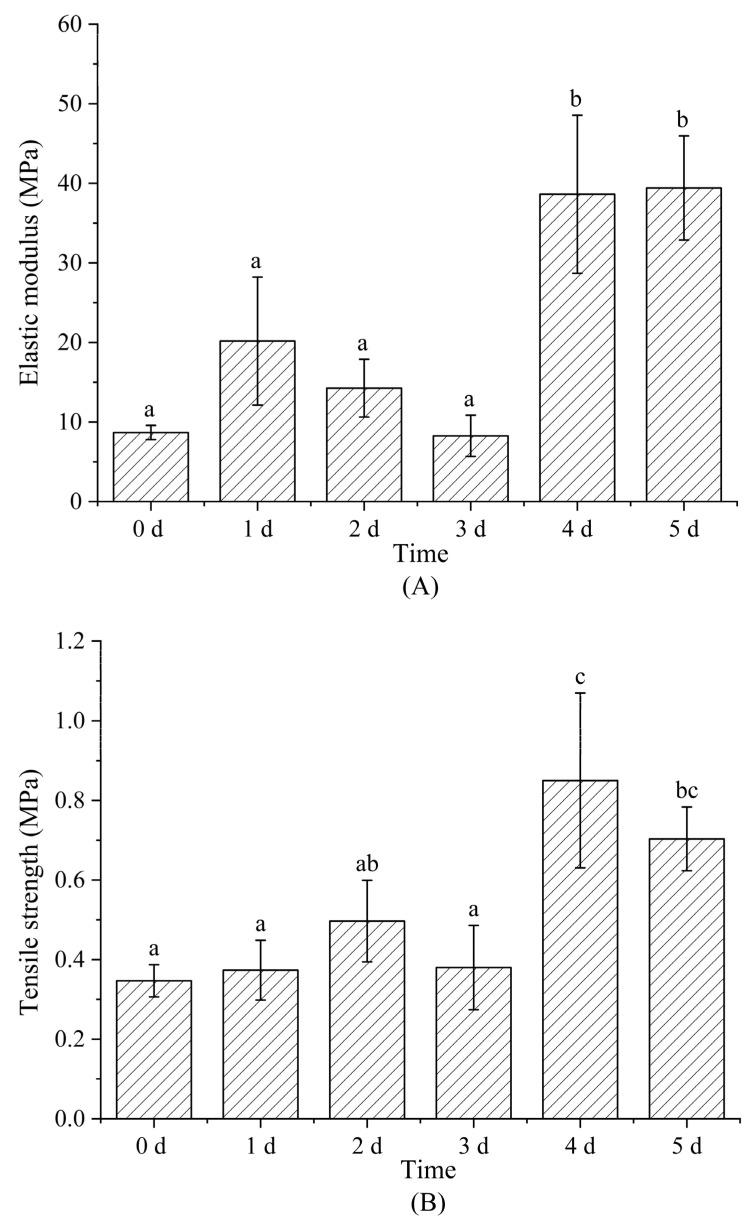

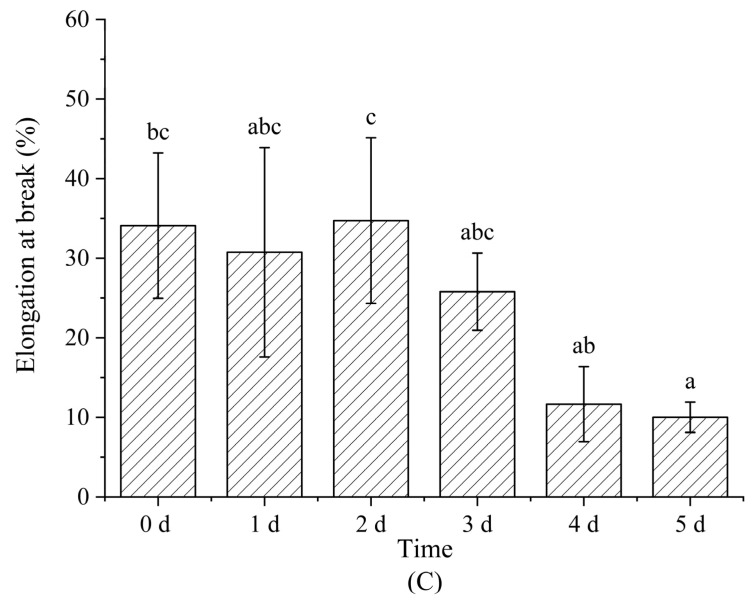
The elastic modulus (**A**), tensile strength (**B**), and elongation at break (**C**) of gelatin/zein/glucose nanofibers after Maillard reactions conducted at 60 °C and 50% RH for 0~5 days. Values with different superscript letters are significantly different (*p* < 0.05).

Figure 10 shows tensile-testing-determined elastic modulus (A), tensile strength (B), and elongation at break (C) of nanofiber films after Maillard reactions conducted for 0–5 d; these parameters reflect nanofiber rigidity, the maximum tensile force maintained in the effective cross-section of the nanofiber prior to breakage, and nanofiber elongation, respectively. Both EM and TS exhibited up-ward trends with increasing reaction time, while EB showed a downward trend. EM, TS, and EB varied from 8.68 ± 0.89 MPa, 0.35 ± 0.04 MPa, and 34.09 ± 9.13% to 39.42 ± 6.54 MPa, 0.70 ± 0.08 MPa, 10.00 ± 1.91%, respectively, over days 1–5. The increases in EM and TS were attributed to the dense structure formed by the protein crosslinking with glucose (the intermolecular entanglements and interactions between polymer chain) [40]. EB was related to the reorientation of nanofibers and the plasticizing effect of glucose in addition to crosslinking [41]. The formation of crosslinked structures and the reduction in glucose plasticization with increasing crosslinking led to lower polymer-chain mobility. Fiber reorientation refers to crosslinking that occurs within and between nanofibers to produce well-aligned and highly ordered, more stable structure [31,42]. Antagonism involving intermolecular forces and fiber forces is required to externally reorient fibers.

### 3.7. Water Vapor Permeability and Water Stability Analysis

**Figure 11:**
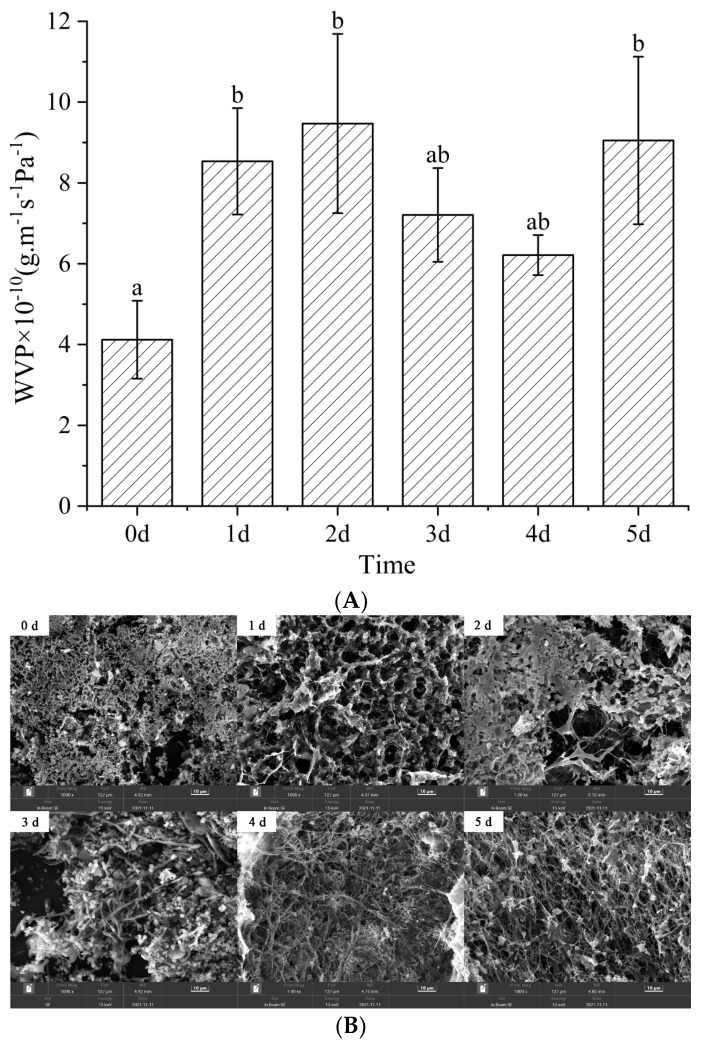
(**A**). Water vapor permeability of gelatin/zein/glucose nanofiber films after Maillard reactions conducted at 60 °C and 50% RH for 0~5 days (values with different superscript letters are significantly different, *p* < 0.05). (**B**). SEM images of gelatin/zein/glucose nanofiber films after immersion in water for 2 months.

The water vapor permeability (WVP) of nanofibers after Maillard reaction for 0~5 days is shown in Figure 11A. WVP was observed to increase significantly during days 0–2, and on day 5, while no significant changes were observed on reaction days 3 and 4. WVP generally involves water vapor absorption and diffusion processes, which are related to the ratio of hydrophilic to hydrophobic components and the arrangement of (polar and non-polar) groups [24,43]. Gelatin and glucose hydrophilia contributed to nanofiber water swelling on day 0, which led to low porosity and the formation of an absorbent mat that exhibited low WVP. However, glycation was complete and the nanofibers were tightly crosslinked after 3~4 days of reaction, which led to lower porosity and a higher number of outward-facing non-polar groups that enhance hydrophobicity and lower the WVP. The production of free amino groups affected WVP by the fifth day, as the reaction entered the intermediate step of the Maillard reaction. This conclusion was consistent with the WCA results.

Figure 11B shows SEM images of nanofiber films immersed in water for two months. Nanofibers swelled with water, fused with each other, and dissolved, which caused the entire fiber structure to collapse. Some fibers and network structures were still evident in the films subjected to the Maillard reaction for 3~5 days. The Maillard reaction resisted the collapse and dissolution of nanofibers in water by improving the network structure and hydrophobicity of nanofibers [14].

## 4. Conclusions

In this study, gelatin/zein nanofibers were successfully fabricated using air-assisted electrospinning, and then glycated with glucose by the Maillard reaction. The significantly higher yield of nanofibers obtained by air-assisted electrospinning highlights its potential as an alternative technology to the traditional electrospinning. The crosslinked nanofiber films exhibited beneficial mechanical strength, hydrophobicity, structural stability, and low color browning after the Maillard reaction under mild conditions, which indicates that natural matrix nanofibers can be modified in a green and cost-effective manner. Therefore, the mild Maillard reaction conditions reported herein offer a promising nanofiber modification method for temperature-sensitive bioactive delivery for the food and biomedical industries.

## Figures and Tables

**Table 1:** The relative absorbance of FTIR spectra of gelatin/zein/glucose nanofibers after Maillard reactions conducted at 60 °C and 50% RH for 0~5 days.

Wavenumber/cm^−1^	3290	2924	1647	1541	1450	1407	1337	1241	1080	1031
0 d	9.17%	5.41%	19.86%	13.71%	8.95%	7.76%	7.15%	7.94%	10.01%	10.04%
1 d	8.41%	4.86%	22.78%	15.05%	9.30%	7.69%	7.05%	7.88%	8.50%	8.47%
2 d	8.61%	6.88%	21.25%	14.40%	9.35%	8.35%	7.03%	7.94%	8.23%	7.95%
3 d	8.35%	6.22%	22.44%	15.01%	9.45%	7.67%	7.11%	7.99%	8.11%	7.65%
4 d	8.27%	5.05%	22.40%	15.17%	9.40%	7.78%	7.15%	7.99%	8.51%	8.28%
5 d	8.22%	5.05%	22.96%	15.28%	9.41%	7.72%	7.10%	7.99%	8.25%	8.03%

**Table 2:** The DSC and TGA data of gelatin/zein/glucose nanofibers after Maillard reactions conducted at 60 °C and 50% RH for 0~5 days.

	DSC		TGA				
	T (°C)	∆H (J/g)	Peak1 (°C)	Weight Loss (%)	Peak2 (°C)	Weight Loss (%)	Residue at 600 °C (%)
0 d	143.5	−3.346	194.4	9.23	313.3	56.29	35.13
1 d	155.4	−4.241	193.3	6.14	318.2	57.13	35.35
2 d	158.6	−4.042	196.6	4.96	319.5	55.25	38.63
3 d	160.8	−5.455	195.6	4.73	305.6	52.12	44.56
4 d	162.7	−4.180	198.5	4.99	315.0	55.75	38.17
5 d	163.9	−5.752	198.9	3.67	313.3	55.14	40.27

## Data Availability

The data are available from the corresponding author.
